# Efficacy of a Web-Based Intervention (Smart Choices 4 Teens) for Facilitating Parent-Adolescent Communication About Relationships and Sexuality: Randomized Controlled Trial

**DOI:** 10.2196/19114

**Published:** 2021-06-15

**Authors:** Beth Bourdeau, Brenda A Miller, Hilary F Byrnes, W Gill Woodall, David B Buller, Joel W Grube

**Affiliations:** 1 Division of Prevention Science University of California San Francisco San Francisco, CA United States; 2 Prevention Research Center Pacific Institute for Research and Evaluation Berkeley, CA United States; 3 Klein Buendel Golden, CO United States

**Keywords:** adolescent, sexual behavior, parenting, intervention, randomized controlled trial, mobile phone

## Abstract

**Background:**

There is a need for interventions that promote healthy decision making among adolescents and leverage the ongoing impact of parental relationships through older adolescence and young adulthood. These interventions should maximize adolescent engagement and be easily accessible to families in terms of cost, duration, and logistics related to participation.

**Objective:**

This study aims to test the efficacy of the healthy relationships and sexual decision-making component of a web-based intervention for older adolescents and their parents, ascertain whether the efficacy varies by gender, and assess its efficacy over time.

**Methods:**

A randomized controlled trial was conducted for the web-based, self-paced intervention Smart Choices 4 Teens from 2014 to 2015. Families (N=411) with adolescents aged 16-17 years were randomly assigned to the intervention or control condition. Surveys assessing aspects of sexual communication were administered at baseline and at 6, 12, and 18 months. Generalized estimating equations were used to determine the impact of exposure to the relationships component of the intervention on sexual communication by parents, as reported by adolescents.

**Results:**

Less than half (88/206, 42.7%) of the intervention group participated in the third and final intervention component, which was focused on relationships and sexual decision making. Participation in the relationships component increased the frequency of parental sexual communication and increased the number of dating rules after accounting for other significant adolescent characteristics. The impact of the intervention varied little by gender, although it did demonstrate an impact on communication reports over the follow-up survey administrations.

**Conclusions:**

Smart Choices 4 Teens demonstrated efficacy in increasing the frequency of sexual communication between parents and adolescents in the long term.

**Trial Registration:**

ClinicalTrials.gov NCT03521115; https://clinicaltrials.gov/ct2/show/NCT03521115

## Introduction

### Background

Adolescents frequently report that parents are their preferred source of information about romantic relationships and sexuality [1,2]. Often, this communication is more focused on daughters [3] than sons and more often initiated by mothers than fathers [4], although fathers’ communication has been shown to increase after participation in interventions [5,6]. Higher amounts of parent-adolescent communication, regardless of topic or other characteristics, are strongly related to increased adolescent safer sex behavior [5,7,8]. Furthermore, the connection between parent-adolescent communication about sex and adolescent sexual behaviors is robust, firmly established [8], and durable through older adolescence and into young adulthood [9-12]. This underscores the importance of encouraging and assisting parents in communicating with their adolescents about sexual decision making.

Recent reviews of interventions designed to facilitate sexual communication between parents and adolescents [6,8,13] indicate that many interventions significantly increased parental levels of comfort and the amount of communication about sex. However, one meta-analysis found that most interventions that address parent-adolescent sexual communication focus on parents of younger adolescents (early adolescents), are often group-based with a trained facilitator, and tend to focus primarily on parents, with very few studies including adolescents [13]. Many of these programs are provided through schools [14,15], medical clinics [16], and community centers [17] and require a substantial time and logistic commitment on the part of participants. Although some programs demonstrate efficacy with their prioritized populations, there is a need to include adolescents within the intervention while extending the reach to a broader audience and more diversified populations for whom time constraints, logistics, and delivery costs present compelling barriers to intervention participation [18].

Digital and web-based delivery platforms [19], including DVDs [20], videogames [21,22], email [23], websites [24], text messages [25,26], and social media [27,28], are increasingly used to address these barriers [29]. These delivery platforms hold promise as most families have computer and internet access, including via smartphones (89%) [30].

### This Study

To summarize, there is a demonstrated need for interventions that support sexual communication between parents and adolescents that (1) support the continued relationship with parents through older adolescence and into emerging adulthood; (2) engage the adolescent in full intervention participation; and (3) address common barriers such as time limitations of participants and reduced cost via delivery in an easily accessible format for families.

This paper aims to examine the adolescent report of sexual and relationship communication outcomes for a web-based intervention, Smart Choices 4 Teens, delivered to parents and their adolescents (aged 16-17 years at enrollment). The program focused on building communication between parents and adolescents on important issues related to adolescent alcohol use, sex, and romantic relationships. The program also provided the skills needed by adolescents to address the social scenarios that they may encounter. Smart Choices 4 Teens was self-paced, on the web only, and contained three components for both parents and adolescents—(1) introduction and parent-adolescent communication skills, (2) adolescent alcohol use, and (3) adolescent relationships and sexuality—and was tested in a randomized controlled trial with 411 parent-adolescent dyads. Results from the alcohol use component have been previously reported [31]; the adolescent relationships and sexuality component is the focus of this paper. Consistent with the theoretical frameworks of the intervention from which it was adapted (see section *Smart Choices 4 Teens Intervention*), we hypothesize that adolescents who had been exposed to the relationships component of the program, compared with those who did not, would report increased sexual communication with their parents (a main effect of intervention exposure) and the increased sexual communication with their parents would vary by gender of the adolescent (interaction effect between intervention exposure and gender).

## Methods

### Participants

**Figure 1:**
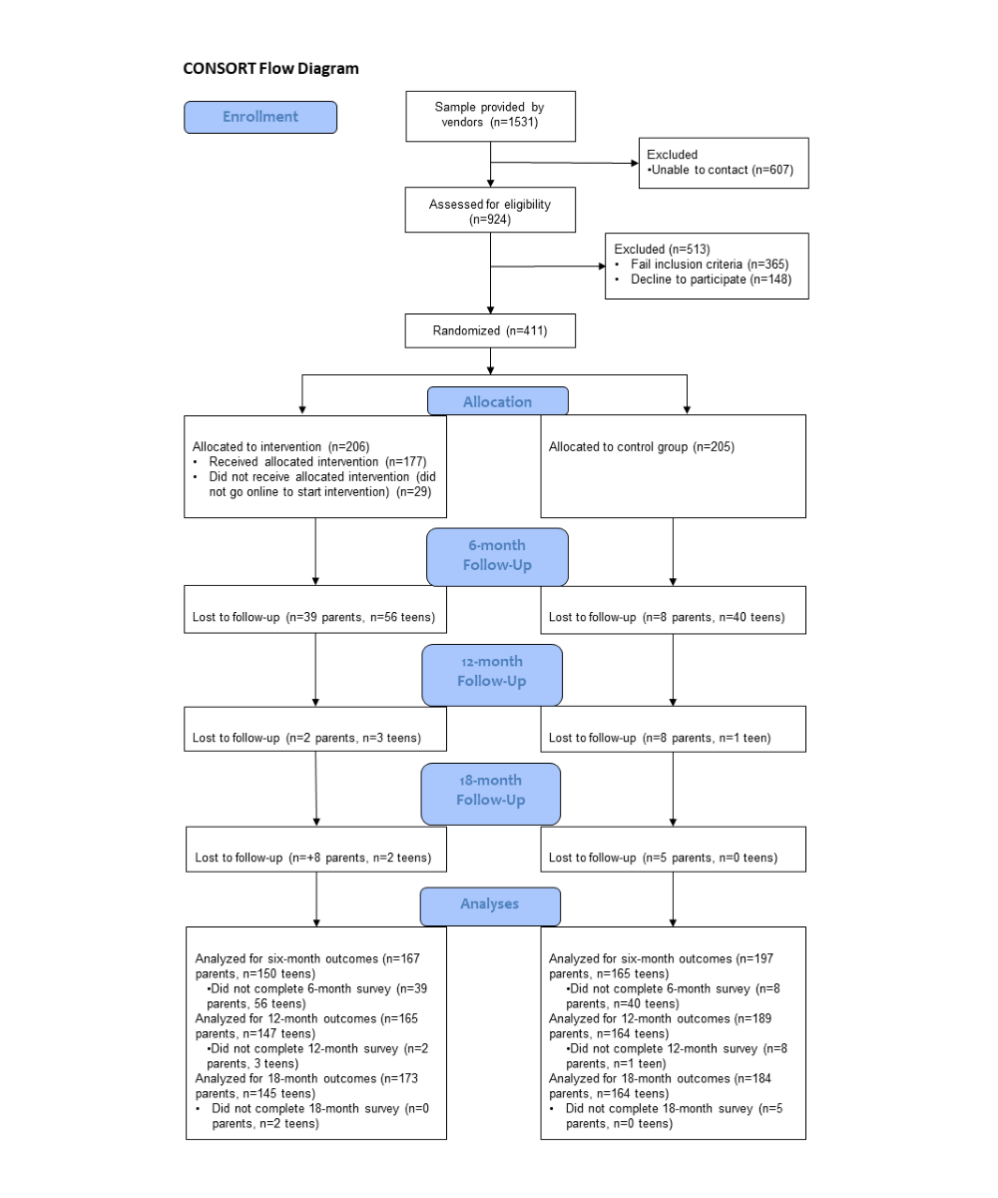
Consolidated Standards of Reporting Trials flow diagram.

Families with adolescents aged 16-17 years were recruited from US-focused web-based panel vendors, companies that matched and recruited participants for targeted survey studies [32], between November 2014 and November 2015 (for an in-depth description of our web-based panel methodology, see the study by Wang-Schweig et al [33]). As shown in the CONSORT (Consolidated Standards of Reporting Trials) diagram (Figure 1 and Multimedia Appendix 1), panel vendors provided contact information for 1531 adult panelists via a secure shared website. Among these, 559 were eligible (ie, parent with an adolescent aged 16-17 years; English speaking; and with a compatible tablet or computer for viewing the web-based intervention). The research team made separate contact with the adolescent to ensure eligibility, ascertain a separate email for the adolescent, and ensure confidentiality. Among the 559 eligible panelists, 411 (73.5%) families completed baseline web-based surveys and were enrolled.

Parents provided consent for themselves and permission for their adolescents, whereas adolescents provided assent for themselves; they were then directed to the research website where they completed separate, confidential baseline surveys. Each participant received US $30 for baseline surveys, US $40 for 6-month follow-up surveys, and US $50 each for 12-month and 18-month follow-up surveys via a mailed check. All study procedures were approved by the Institutional Review Board of the Pacific Institute for Research and Evaluation.

Using a 1:1 allocation ratio and a computer-generated program, parent and adolescent dyads were randomly assigned to either the intervention (n=206) or control (n=205) condition as a randomized controlled trial without a waitlist control group design. Dyads in both conditions received links to resources, including hotline numbers and websites providing information about adolescent alcohol and drug use, sexual behavior, suicide, support for gender and sexual minority adolescents, and other health issues. All families also had access to an 800 number throughout the duration of the project for contact with the research team. Automated emails and texts were sent to invite families to complete the follow-up surveys at 6, 12, and 18 months after baseline. One week after the follow-up survey, invitations were sent and reminder emails or texts were sent to participants who had not completed the surveys, with the emails or texts repeated after another week of nonresponse. If not completed within 2 weeks, phone calls were made to the participants to encourage completion.

### Smart Choices 4 Teens Intervention

The intervention was targeted to older adolescents (aged 16-17 years) not as sex education but to address decision making and skills needed in the context of increased independence and sensitivity to the needs of adolescents who may have initiated some alcohol use and sexual behavior. The intervention sought to increase parent-adolescent communication and build skills to address scenarios that adolescents encounter related to alcohol use and romantic relationships. Facilitation and clarification of adolescent choices were guided by an offline discussion of scenarios derived from real stories that require decision making by adolescents. These discussions offered opportunities for the values of the parents and adolescents to be considered. Specifically, what constituted smart decision making was determined by the families and not asserted by the intervention.

Smart Choices 4 Teens was created by adapting materials from two evidence-based prevention programs that are both booklet-based and independently completed by families: *Family Matters* [34] (designed for parents of younger adolescents) and *A Parent Handbook for Talking with College Students about Alcohol* [35] (designed for parents to prepare their adolescents for the first year as college students). *Family Matters* addresses theoretically derived risk and protective factors important to adolescent alcohol and other drug use and emphasizes parental characteristics (eg, supervision, support, communication skills, attachment, and conflict resolution skills) that can be strengthened to influence adolescents’ risky behaviors. Strengthening adolescent characteristics (eg, educational achievement, communication, conflict resolution skills, and response to peer pressure) in the context of the parent-adolescent relationship subsequently creates resilience in adolescents and decreases risky behaviors. These characteristics are central to theories of socialization [36,37], social control [38,39], social development [40], and family interaction [41]. Content for the program is based on social learning and communication theories [42-45] and is delivered in booklet form to be delivered by parents to adolescents. *Parent Handbook* is designed for parents to provide practical approaches to address alcohol use among adolescents and is designed to address problems for adolescents making the transition from high school to college. On the basis of a theoretical approach that takes into account the importance of social, cognitive (eg, beliefs and social norms), emotional, and decision-making aspects of college drinking, *Parent Handbook* is effective in reducing drinking and drinking-related consequences, influences perceptions of drinking activities, and impacts perceived parental and peer approval of drinking [46]. Both programs have evidence of efficacy and effectiveness and were adapted for the following considerations: (1) age appropriateness for those aged 16-17 years; (2) parallel materials for the adolescent; and (3) delivery of the materials to a web-based platform. The adaptation process included creating instructional objectives, content outline, activity descriptions, and scripts through a series of iterative development cycles that included review by an expert panel and input from two rounds of focus groups with parents and adolescents. Additional information on the adaptation of the interventions is available with the results for the alcohol component [31].

For each component, Smart Choices 4 Teens required that parents and adolescents complete web-based materials separately. Following completion, they chose two of four possible scenarios for discussion. These scenarios were designed to use the skills and knowledge addressed in the component activities. After the discussion was completed, the participants moved to the next component. Given the imposed chronological order of the components, families first completed the communications (parents: mean 28.13, SD 22.39 min; adolescents: mean 23.45, SD 24.04 min) and alcohol (parents: mean 18.75, SD 24.20 min; adolescents: mean 18.63, SD 25.21 min) components before moving forward with the relationships component. The average time spent on the relationships component was nearly identical between parents (mean 23.53, SD 32.37 min) and adolescents (mean 24.56, SD 35.11 min). Families typically completed the intervention over several weeks.

The goal of the relationships component was to foster communication around healthy relationships [47], increase parental monitoring [48-50], clarify expectations and values, provide skills for making healthy choices around the decisions related to relationships, and encourage reflection on decision making. This component was divided into five sections and was delivered via a video narrative, infogadget (activity with a series of tabs on a single topic, containing graphics and text), and interactive activity: (1) acknowledging how challenging parent-adolescent communication about relationships and sexuality can be (video narrative); (2) reflecting on healthy relationships (readiness for a relationship [interactive activity], prioritizing partner characteristics [interactive activity], and signs of emotional and verbal abuse [video narrative]); (3) smart decision making and sex expectancies (interactive activity), unintended health consequences such as pregnancy and sexually transmitted infections (infogadget), social media responsibility (interactive activity), and refusal skills (video examples); and (4) values and guidelines (interactive activity).

Following the completion of the web-based materials, the families were asked to engage in an offline discussion, guided by four real-life scenarios pertaining to the complexities of relational decision making, which were drawn from focus groups with adolescents for another adaptation of *Family Matters* [51]. The scenarios were presented as dilemmas facing an adolescent and created an opportunity for a dialog about the options that the hypothetical adolescent in the story had for addressing the dilemma. The four scenarios included (1) facing a situation in which adolescents were deciding whether to have sex within the context of a relationship; (2) decision making about relationships in the context of other friendships (dating a friend’s sibling); (3) an adolescent who encountered a friend who had passed out from alcohol use and was possibly vulnerable to sexual or physical assault; and (4) relationships between individuals with large age discrepancies (adolescent girls and older men). Families selected two scenarios to discuss offline and were prompted to download a tailored discussion guide for the parent-adolescent discussion. Given the normative increase in sexual exploration of older adolescents and an imperative to protect their privacy and potential safety, activities requiring communication between the dyads were designed so that self-disclosure on the part of the adolescents was not required (any sexual behaviors, sexual orientation, etc).

### Measures

The following naming convention is used for reporting on the waves’ descriptive and reliability statistics: baseline (T0) and follow-ups for 6 months (T1), 12 months (T2), and 18 months (T3). For all measures, baseline assessments were phrased to indicate a general (or *ever*) assessment, whereas the follow-up surveys explicitly asked for an assessment within the past 6 months or since the previous survey.

### Primary Outcomes: Parent-Adolescent Communication

#### Overall Sex Communication Frequency

The overall frequency of sex communication was assessed with a single item adapted from the evaluation of the program Parent Handbook for Talking with College Students about Alcohol [52]—“Overall, how often have you talked to your parent about sex”—with response options ranging from (1) *never* through (5) *very often*.

#### Topic Specific Communication Frequency

Teens reported the frequency of ten specific parent-teen sexual communication topics [41] (‘How often have you and your parent talked about your having sexual intercourse and…’) with responses ranging from ‘never’ (1) to ‘very often’ (5). Topics included ‘the negative impact on your social life because you would lose the respect of others’, ‘the importance of being committed to a healthy lifestyle and how being in a sexual relationship can impact this’, ‘how embarrassing it would be if I caught an STI (sexually transmitted infection)’. The mean score of these items was used for the analyses (T0: α =.92; T1 α =.94; T2: α =.94; T3: α =.94).

#### Topics of Conversation About Sex

Adolescents were asked to report whether their parents had talked about specific topics with them, adapted from the evaluation of *Family Matters* [53]. Items were summed for one total score and summed by topic area as detailed below.

#### Delaying Sex

Three items asked whether a parent had cautioned the adolescent not to have sex, not to have a serious relationship in high school, and not to have sex specifically because their religion or values forbid sex outside of marriage. A sum of the three items was taken, and reliability was adequate (T0: α=.62; T1: α=.64; T2: α=.68; T3: α=.70).

#### Health Risks

Two items were summed for discussions of health risks: “sex can result in pregnancy” and “sex can result in a sexually transmitted infection.” The two items were significantly correlated for all four waves (T0: *r*^2^=0.58; *P*<.001; T1: *r*^2^=0.71; *P*<.001; T2: *r*^2^=0.74; *P*<.001; T3: *r*^2^=0.77; *P*<.001).

#### Birth Control

A single item asked, “if you are sexually active, [your parent(s)] will provide birth control.”

#### Sex Permissive

For adolescents, two items were summed to create a “sex permissive message” including “being sexually active is okay” and “under what circumstances, if any, it is ok to be sexually active.” The two items were significantly correlated at all four waves (T0: *r*^2^=0.49; *P*<.001; T1: *r*^2^=0.54; *P*<.001; T2: *r*^2^=0.51; *P*<.001; T3: *r*^2^=0.42; *P*<.001).

#### Monitoring and Dating Rules

Adolescents were each asked about the expectations for behavior in romantic relationships that they had decided upon [54]. They were asked to respond no (0) or yes (1) to a list of 15 possible rules, for example, “no dates on school nights,” “come home at an agreed-upon time (curfew),” “use good judgment,” and “be a gentleman/lady.” Responses were summed. Internal consistency was good (T0: α=.83; T1: α=.87; T2: α=.86; T3: α=.89).

### Program Participation

A measure of program participation was captured in two dichotomous variables—one for those participants who only engaged with the communication and alcohol components (0 all others) and one for those who engaged with some or all of the relationships component (0 all others). Thus, participants in the control group and those in the intervention group who did not initiate the program were the referents for the analyses.

### Controls

#### Overview

The following five baseline adolescent characteristics were included: gender, age, race or ethnicity, sexual orientation, and sexual experience. Gender was dichotomized as female (0) and male (1), and age was treated as a continuous variable. Participants were encouraged to choose all racial or ethnic backgrounds that applied to them (American Indian or Alaska Native, Asian, Black of African American, Native Hawaiian or other Pacific Islander, White, and Other) and asked separately if they identify as Latino or Hispanic. Race or ethnicity was dichotomized, with White non-Hispanic coded as 0 and any affirmative racial or ethnic minority coded as 1. The measure of sexual identity followed best practice for assessing sexual orientation [55] as a composite of several responses, as follows: how they identify (heterosexual or straight, gay or lesbian, bisexual, or other), to whom they are attracted (only females, mostly females, equally males and females, mostly males, or only males), and with whom they have had sexual contact (males only, females only, both, or neither) [56]. Using their reported gender identity, participants were considered sexual minorities if they (1) chose a nonheterosexual self-identification, (2) reported any same-sex sexual contact, or (3) reported any attraction to the same sex. This was recoded into one final item indicating heterosexual (357/411, 74.9%; coded as 0) and sexual (79/411, 19.2%; coded as 1) minorities. Finally, adolescents’ responses to the question “Have you ever had sex (vaginal, oral, or anal)?” was coded such that negative responses were 0 and affirmative responses were 1.

#### Selection Model

As parents and adolescents could choose how far to continue with the program, selection bias may have confounded the dosage analyses. One approach to help account for such biases is to model the selection process and then include an instrumental variable representing it as a predictor in the primary analyses [57]. In this case, we conducted a probit analysis to predict the completion of the relationships component from relevant baseline measures and calculated an inverse Mills ratio (IMR) for each respondent based on the probit model. The IMR (nonselection hazard) was calculated in Stata version 15 (StataCorp LLC) using the two-step procedure described by Heckman [57]. This ratio is a function of the predicted probability (propensity) of completing the component and represents the underlying selection process. Given the focus of the relationships component, the baseline predictors included measures of parents’ and adolescents’ evaluations of and levels of communication in their relationship, parent-adolescent trust, parental monitoring, sexual communication, and demographics. Overall communication, adolescents’ reports of ever having sex, gender, sexual orientation, and age were included in the selection models. Parents’ self-reports of gender, income, and ethnicity were also used. When the IMR is significant in a model, this indicates that the predicted probability of completing the component is associated with the outcome, that is, the same factors that predispose families to complete (or not complete) the components are related to the outcomes. In models where program exposure is significant, this indicates that even accounting for factors that predict program completion, participation in the intervention is still related to outcomes. Selection models provide several benefits, including ease of use and wide use in research (a 700% increase in use over the last decade [58,59]). Selection models also address the selection of unobservable factors, whereas alternatives such as propensity score matching require self-selection of participants to be explained completely by observable factors [59].

### Analyses

Examining alternative models, such as dosage models, has been proposed for trials with limited control over program exposure and where substantial portions of participants do not appear motivated to fully adhere to the intervention implementation protocol [60]. Participants may lack motivation to complete prevention programs because the illnesses or discomforts have not yet occurred [60,61] in comparison with treatment trials that typically aim to improve an existing problem or condition (eg, alcoholism or diabetes). High rates of nonadherence in prevention trials could introduce type II errors by underestimating the effectiveness of the intervention [60]. For these reasons, we examined outcomes for experimental participants based on the level of program exposure, testing the hypothesis that some program exposure, more so relationships component exposure, will be related to increased sexual communication.

Outcomes were assessed using generalized estimating equations in SPSS (version 25) to account for repeated measures of each outcome (inclusive of baseline report) nested within participants. In each analysis, variables included adolescent gender, age, ethnicity, sexual minority status, report of sexual experience at baseline, measurement period, the IMR, and the two dichotomous variables for exposure to the intervention (one or two components: communications and alcohol only; all three components: communications, alcohol, and relationships component), such that those with no exposure comprised the referent group. The analysis of hypothesis 1 included only tests of the main effects of the variables. The analysis of hypothesis 2 added an interaction term for gender based on exposure to the intervention.

## Results

### Overview

At baseline, adolescents were aged 16-17 (mean 16.4, SD 0.5) years, and slightly more than half (226/411, 55.3%) of them were girls. About one-tenth (38/411, 9.5%) of the adolescents were Hispanic or Latino. Adolescents reported the following race or ethnicities: 72.5% (298/411) White, 1.9% (8/411) Asian, 11.7% (48/411) African American, 1% (4/411) Native American, 8.3% (34/411) multiracial, 2.7% (11/411) some other race, and 1.9% (8/411) unreported. There were no significant differences in demographic characteristics between the experimental and control conditions or among the amounts of intervention dosage. Table 1 presents intervention dosage and survey completion rates.

**Table 1:** Intervention dosage and survey completion (N=411).

Survey wave or intervention component			Intervention (n=206), n (%)		Control (n=205), n (%)
Baseline (T0) survey completion			206 (100)		205 (100)
**Intervention exposure**					
	No exposure	29 (14.1)		—^a^	
	Communication or communication and alcohol	89 (43.2)		—	
	Communication, alcohol, and relationships	88 (42.7)		—	
6-month (T1) survey completion			150 (72.8)		165 (80.5)
12-month (T2) survey completion			147 (71.4)		164 (80)
18-month (T3) survey completion			145 (70.4)		164 (80)

^a^Not available; those in the control group were not permitted to participate in the intervention and thus have no exposure data.

### Changes Over Four Survey Waves (Baseline Through 18-Month Follow-up)

For all adolescents regardless of randomization or program exposure, there was a significant main effect of time on most communication outcomes, with most reports by adolescents decreasing over time (data not shown). There were significant decreases over time for overall frequency of communication, average frequency of specific topics, and number of topics discussed. There were also significant decreases in conversations regarding specific topics, including delaying sex, health risks, and dating rules. In contrast, there was a significant increase in conversations reflecting sex permissiveness at 12 months and again at 18 months. Discussions related to parental provision of birth control showed no main effects across time.

### Adolescent Characteristics

Gender had a significant effect on most sexual communication outcomes. Adolescent girls reported greater sexual communication with parents regarding overall frequency, average topic frequency, and number of topics and were more likely to report communicating with parents about delaying sex. They were also more likely to report a greater number of dating rules. Boys were more likely than girls to report communication indicating that parents would provide birth control. There were no differences between genders regarding communication about sex permissiveness or health risks.

Previous sexual experience at baseline had a significant main effect on reported sexual communication with parents. Adolescents who reported previous sexual experience also had greater reports of overall frequency, average frequency, and number of topics discussed with parents. Adolescents with previous sexual experiences also reported greater sex permissiveness, health risk communication, and fewer reports of birth control provision, delaying sex, and dating rules.

Adolescents’ sexual minority status also had significant main effects, with those whose sexual orientation was not strictly heterosexual, reporting more sexual permissive communications with parents. They also reported a lower average frequency of communication, less discussion about delaying sex, and fewer dating rules.

A few differences were found between White and racial or ethnic minority adolescents with White adolescents reporting greater average frequency and more discussions on delaying sex.

### Hypothesis 1: Main Effects of Relationships Component

Significant main effects were found for participating in the intervention after controlling for gender, racial or ethnic status, age at baseline, sexual minority status, sexual experience, the IMR, and time in the model (Tables 2-4). As hypothesized, adolescents engaged in the relationships component reported higher levels of overall frequency of sexual communication with their parents (B*=*0.30; SE 0.11; 95% CI 0.08-0.52; *P*=.007) than those with no exposure and those who participated in only the communication or alcohol component. Adolescents exposed to the relationships component reported significantly higher average topic frequency (B*=*0.27; SE 0.12; 95% CI 0.03-0.51; *P*=.03) and a greater number of dating rules (B*=*0.72; SE 0.37; 95% CI 0-1.44; *P*=.049). There were no main effects on other communication outcomes. As noted earlier, those who were exposed to only the communications and alcohol components were considered separately from those who were exposed to the relationships component. Exposure to only the communications or alcohol components was significantly associated with lower average topic frequency (B*=*−0.24; SE 0.12; 95% CI −0.47 to −0.01; *P*=.04).

**Table 2:** Results from generalized estimating equations: main effects of intervention exposure on adolescent report of sexual communication: topics (T0: n=411; T1: n=325; T2: n=311; T3: n=309).

Variables	Overall frequency					Average topic frequency					Number of topics			
	B	SE	95% CI	*P* value	B		SE	95% CI	*P* value	B		SE	95% CI	*P* value
Intercept	2.455	3.082	−3.585 to 8.495	.43	1.825		3.126	−4.301 to 7.951	.56	4.527		5.317	−5.895 to 14.948	.39
18 months	−0.437	0.069	−0.572 to −0.302	<.001	−0.436		0.057	−0.547 to −0.324	<.001	−0.690		0.116	−0.917 to −0.462	<.001
12 months	−0.352	0.065	−0.479 to −0.225	<.001	−0.358		0.048	−0.452 to −0.264	<.001	−0.424		0.102	−0.624 to −0.224	<.001
6 months	−0.360	0.066	−0.490 to −0.231	<.001	−0.344		0.051	−0.444 to −0.243	<.001	−0.638		0.107	−0.848 to −0.427	<.001
Male gender	−0.291	0.093	−0.473 to −0.108	.002	−0.254		0.095	−0.441 to −0.067	.008	−0.507		0.161	−0.823 to −0.191	.002
Sexual minority	0.020	0.133	−0.240 to 0.280	.88	−0.268		0.129	−0.520 to −0.016	.04	−0.053		0.200	−0.445 to 0.338	.79
Sexual experience	0.402	0.107	0.192 to 0.612	<.001	0.364		0.108	0.152 to 0.576	.001	0.712		0.173	0.374 to 1.051	<.001
Age (years)	−0.087	0.096	−0.274 to 0.101	.37	0.008		0.098	−0.183 to 0.200	.93	−0.025		0.165	−0.349 to 0.299	.88
Racial or ethnic minority	−0.107	0.113	−0.329 to 0.115	.34	−0.217		0.113	−0.439 to 0.005	.06	−0.294		0.178	−0.643 to 0.055	.09
Inverse Mills ratio	1.891	2.969	−3.928 to 7.710	.52	1.081		2.909	−4.622 to 6.783	.71	0.274		4.836	−9.205 to 9.752	.96
Exposure to communications or alcohol component	−0.139	0.119	−0.372 to 0.094	.24	−0.238		0.116	−0.465 to −0.011	.04	−0.283		0.208	−0.690 to 0.124	.17
Exposure to relationships component	0.299	0.111	0.081 to 0.516	.01	0.268		0.122	0.029 to 0.507	.03	0.132		0.174	−0.210 to 0.474	.45

**Table 3:** Results from generalized estimating equations: main effects of intervention exposure on adolescent report of sexual communication: cautionary communication (T0: n=411; T1: n=325; T2: n=311; T3: n=309).

Variables	Delay sex					Health risks					Dating rules			
	B	SE	95% CI	*P* value	B		SE	95% CI	*P* value	B		SE	95% CI	*P* value
Intercept	2.657	3.208	−3.630 to 8.943	.41	2.077		1.597	−1.052 to 5.206	.19	27.295		10.318	7.072 to 47.518	.008
18 months	−0.382	0.058	−0.495 to −0.269	<.001	−0.422		0.050	−0.520 to −0.323	<.001	−1.245		0.174	−1.586 to −0.903	<.001
12 months	−0.263	0.054	−0.369 to −0.157	<.001	−0.280		0.045	−0.368 to −0.191	<.001	−0.680		0.157	−0.988 to −0.372	<.001
6 months	−0.333	0.054	−0.437 to −0.228	<.001	−0.312		0.046	−0.401 to −0.223	<.001	−0.502		0.163	−0.822 to −0.181	.002
Male gender	−0.326	0.097	−0.517 to −0.135	.001	−0.082		0.058	−0.196 to 0.032	.16	−1.282		0.318	−1.906 to −0.658	<.001
Sexual minority	−0.384	0.117	−0.613 to −0.154	.001	−0.041		0.069	−0.177 to 0.094	.55	−1.088		0.426	−1.923 to−0.252	.01
Sexual experience	−0.219	0.103	−0.420 to −0.018	.03	0.182		0.059	0.067 to 0.297	.002	−1.249		0.346	−1.928 to−0.569	<.001
Age (years)	−0.106	0.096	−0.293 to 0.082	.27	0.006		0.058	−0.108 to 0.121	.91	−0.151		0.311	−0.761 to 0.458	.63
Racial or ethnic minority	−0.276	0.110	−0.492 to −0.060	.01	−0.092		0.063	−0.216 to 0.032	.15	−0.061		0.359	−0.764 to 0.641	.86
Inverse Mills ratio	0.967	2.943	−4.802 to 6.736	.74	−0.327		1.549	−3.362 to 2.709	.83	−14.403		10.268	−34.528 to 5.722	.16
Exposure to communications or alcohol component	0	0.119	−0.234 to 0.234	.99	−0.101		0.077	−0.251 to 0.049	.19	0.066		0.368	−0.655 to 0.787	.86
Exposure to relationships component	0.075	0.114	−0.149 to 0.298	.51	0.051		0.065	−0.075 to 0.178	.43	0.720		0.365	0.004 to 1.435	.05

**Table 4:** Results from generalized estimating equations: main effects of intervention exposure on adolescent report of sexual communication: positive communication (T0: n=411; T1: n=325; T2: n=311; T3: n=309).

Variables	Provide birth control					Sex permissive			
	B	SE	95% CI	*P* value	B		SE	95% CI	*P* value
Intercept	11.322	6.018	−0.472 to 23.117	.06	1.193		1.889	−2.509 to 4.895	.53
18 months	−0.047	0.145	−0.331 to 0.237	.75	0.112		0.050	0.014 to 0.210	.03
12 months	0.037	0.125	−0.208 to 0.281	.77	0.105		0.046	0.015 to 0.195	.02
6 months	0.067	0.129	−0.187 to 0.320	.61	0.019		0.049	−0.076 to 0.115	.69
Male gender	0.885	0.189	0.516 to 1.255	<.001	0.071		0.059	−0.045 to 0.187	.23
Sexual minority	−0.334	0.242	−0.809 to 0.140	.17	0.272		0.086	0.103 to 0.440	.002
Sexual experience	−1.405	0.213	−1.823 to −0.988	<.001	0.441		0.075	0.294 to 0.588	<.001
Age (years)	−0.104	0.196	−0.488 to 0.280	.59	0.051		0.063	−0.072 to 0.175	.41
Racial or ethnic minority	−0.247	0.217	−0.672 to 0.179	.26	0.027		0.066	−0.102 to 0.157	.68
Inverse Mills ratio	−10.420	5.826	−21.837 to 0.998	.07	−1.924		1.898	−5.644 to 1.795	.31
Exposure to communications or alcohol component	0.414	0.232	−0.041 to 0.869	.07	−0.097		0.072	−0.239 to 0.045	.18
Exposure to relationships component	−0.153	0.222	−0.588 to 0.282	.49	−0.019		0.071	−0.159 to 0.120	.78

### Hypothesis 2: Interaction Effects of the Intervention by Gender

To assess the impact of exposure to the relationships component by gender, a series of analyses was conducted with interaction terms (Tables 5-7). The findings suggest that the impact of the intervention was stronger for girls than boys for overall frequency of sexual communication (B*=*−0.41; SE 0.21; 95% CI −0.81 to −0.01; *P*=.046). However, the significant main effect of the intervention did not vary by gender for average frequency of specified sexual topics (relationships component: *P*=.01; relationships component by gender: *P*=.12) or for dating rules (relationships component: *P*=.01; relationships component by gender: *P*=.32).

**Table 5:** Results from generalized estimating equations: interaction effects of intervention exposure on adolescent report of sexual communication by gender: topics (T0: n=411; T1: n=325; T2: n=311; T3: n=309).

Variables	Overall frequency					Average topic frequency					Number of topics			
	B	SE	95% CI	*P* value	B		SE	95% CI	*P* value	B		SE	95% CI	*P* value
Intercept	2.277	3.077	−3.755 to 8.309	.46	1.691		3.105	−4.396 to 7.777	.59	4.404		5.306	−5.995 to 14.803	.41
18 months	−0.437	0.069	−0.572 to −0.302	<.001	−0.436		0.057	−0.547 to −0.324	<.001	−0.689		0.116	−0.917 to −0.462	<.001
12 months	−0.352	0.065	−0.479 to −0.225	<.001	−0.358		0.048	−0.451 to −0.264	<.001	−0.424		0.102	−0.624 to −0.224	<.001
6 months	−0.360	0.066	−0.490 to −0.230	<.001	−0.344		0.051	−0.444 to −0.243	<.001	−0.637		0.107	−0.848 to −0.427	<.001
Male gender	−0.200	0.109	−0.414 to 0.014	.07	−0.175		0.108	−0.387 to 0.037	.11	−0.438		0.189	−0.808 to −0.069	.02
Sexual minority	0.033	0.133	−0.228 to 0.295	.80	−0.256		0.129	−0.509 to −0.003	.05	−0.043		0.201	−0.437 to 0.350	.83
Sexual experience	0.396	0.106	0.189 to 0.604	<.001	0.359		0.108	0.148 to 0.571	.001	0.708		0.173	0.369 to 1.047	<.001
Age	−0.085	0.095	−0.272 to 0.102	.37	0.008		0.098	−0.183 to 0.200	.93	−0.024		0.165	−0.348 to 0.299	.88
Racial or ethnic minority	−0.119	0.115	−0.344 to 0.105	.29	−0.228		0.114	−0.452 to −0.005	.05	−0.304		0.179	−0.654 to 0.046	.09
Inverse Mills ratio	2.037	2.960	−3.765 to 7.839	.49	1.201		2.887	−4.458 to 6.860	.68	0.377		4.837	−9.103 to 9.857	.94
Exposure to communications or alcohol component	−0.145	0.119	−0.378 to 0.088	.22	−0.243		0.116	−0.471 to −0.016	.04	−0.287		0.208	−0.695 to 0.121	.17
Exposure to relationships component	0.462	0.153	0.161 to 0.763	.003	0.414		0.167	0.086 to 0.742	.01	0.255		0.214	−0.164 to 0.675	.23
Exposure to relationships by gender	−0.408	0.205	−0.809 to −0.006	.05	−0.359		0.230	−0.809 to 0.091	.12	−0.306		0.342	−0.975 to 0.364	.37

**Table 6:** Results from generalized estimating equations: interaction effects of intervention exposure on adolescent report of sexual communication by gender: cautionary communication (T0: n=411; T1: n=325; T2: n=311; T3: n=309).

Characteristics	Delay sex						Health risks					Dating rules			
	B	SE	95% CI		*P* value	B		SE	95% CI	*P* value	B		SE	95% CI	*P* value
Intercept	2.630	3.205		−3.652 to 8.912	.41	1.978		1.597	−1.152 to 5.108	.22	27.001		10.275	6.864 to 47.139	.009
18 months	−0.382	0.058		−0.495 to −0.269	<.001	−0.422		0.050	−0.520 to −0.323	<.001	−1.244		0.174	−1.586 to −0.902	<.001
12 months	−0.263	0.054		−0.369 to −0.157	<.001	−0.279		0.045	−0.368 to −0.190	<.001	−0.680		0.157	−0.988 to −0.372	<.001
6 months	−0.332	0.054		−0.437 to −0.228	<.001	−0.311		0.046	−0.401 to −0.222	<.001	−0.501		0.163	−0.821 to −0.180	.002
Male gender	−0.310	0.110		−0.526 to −0.094	.005	−0.029		0.068	−0.162 to 0.103	.67	−1.117		0.360	−1.822 to −0.412	.002
Sexual minority	−0.381	0.118		−0.612 to −0.151	.001	−0.034		0.071	−0.173 to 0.105	.63	−1.063		0.425	−1.896 to −0.229	.01
Sexual experience	−0.220	0.103		−0.421 to −0.018	.03	0.178		0.059	0.063 to 0.294	.003	−1.258		0.347	−1.938 to −0.578	<.001
Age	−0.105	0.096		−0.293 to 0.082	.27	0.007		0.058	−0.107 to 0.121	.90	−0.151		0.311	−0.760 to 0.459	.63
Racial or ethnic minority	−0.278	0.109		−0.493 to −0.064	.01	−0.100		0.063	−0.223 to 0.024	.11	−0.085		0.355	−0.782 to 0.612	.81
Inverse Mills ratio	0.990	2.945		−4.782 to 6.762	.73	−0.245		1.557	−3.296 to 2.806	.88	−14.142		10.266	−34.263 to 5.979	.17
Exposure to communications or alcohol component	−0.001	0.119		−0.235 to 0.233	.99	−0.104		0.077	−0.255 to 0.047	.18	0.055		0.369	-0.668 to 0.777	.88
Exposure to relationships component	0.103	0.140		−0.171 to 0.377	.46	0.144		0.077	−0.008 to 0.296	.03	1.023		0.400	0.239 to 1.808	.01
Exposure to relationships by gender	−0.070	0.228		−0.517 to 0.377	.76	−0.230		0.130	−0.484 to 0.023	.08	−0.751		0.747	−2.216 to 0.713	.32

**Table 7:** Results from generalized estimating equations: interaction effects of intervention exposure on adolescent report of sexual communication by gender: positive communication (T0: n=411; T1: n=325; T2: n=311; T3: n=309).

Variables	Provide birth control					Sex permissive			
	B	SE	95% CI	*P* value	B		SE	95% CI	*P* value
Intercept	11.497	6.025	−0.312 to 23.307	.06	1.234		1.888	−2.468 to 4.935	.51
18 months	−0.046	0.145	−0.331 to 0.239	.75	0.112		0.050	0.014 to 0.210	.03
12 months	0.036	0.125	−0.209 to 0.282	.77	0.105		0.046	0.015 to 0.195	.02
6 months	0.067	0.130	−0.188 to 0.321	.61	0.019		0.049	−0.076 to 0.115	.69
Male gender	0.788	0.220	0.357 to 1.220	<.001	0.052		0.069	−0.083 to 0.187	.45
Sexual minority	−0.348	0.244	−0.827 to 0.131	.16	0.269		0.086	0.100 to 0.439	.002
Sexual experience	−1.399	0.214	−1.819 to −0.979	<.001	0.442		0.075	0.295 to 0.589	<.001
Age	−0.104	0.196	−0.489 to 0.280	.59	0.051		0.063	−0.072 to 0.174	.42
Racial or ethnic minority	−0.235	0.219	−0.664 to 0.194	.28	0.030		0.066	−0.099 to 0.159	.65
Inverse Mills ratio	−10.580	5.814	−21.976 to 0.816	.07	−1.960		1.897	−5.679 to 1.759	.30
Exposure to communications or alcohol component	0.419	0.231	−0.033 to 0.871	.07	−0.096		0.072	−0.238 to 0.046	.19
Exposure to relationships component	−0.308	0.288	−0.872 to 0.255	.28	−0.054		0.093	−0.237 to 0.128	.56
Exposure to relationships by gender	0.431	0.435	−0.421 to 1.283	.32	0.087		0.135	−0.178 to 0.351	.52

### Post Hoc: Interaction Effects of the Intervention Over Time by Gender

**Figure 2:**
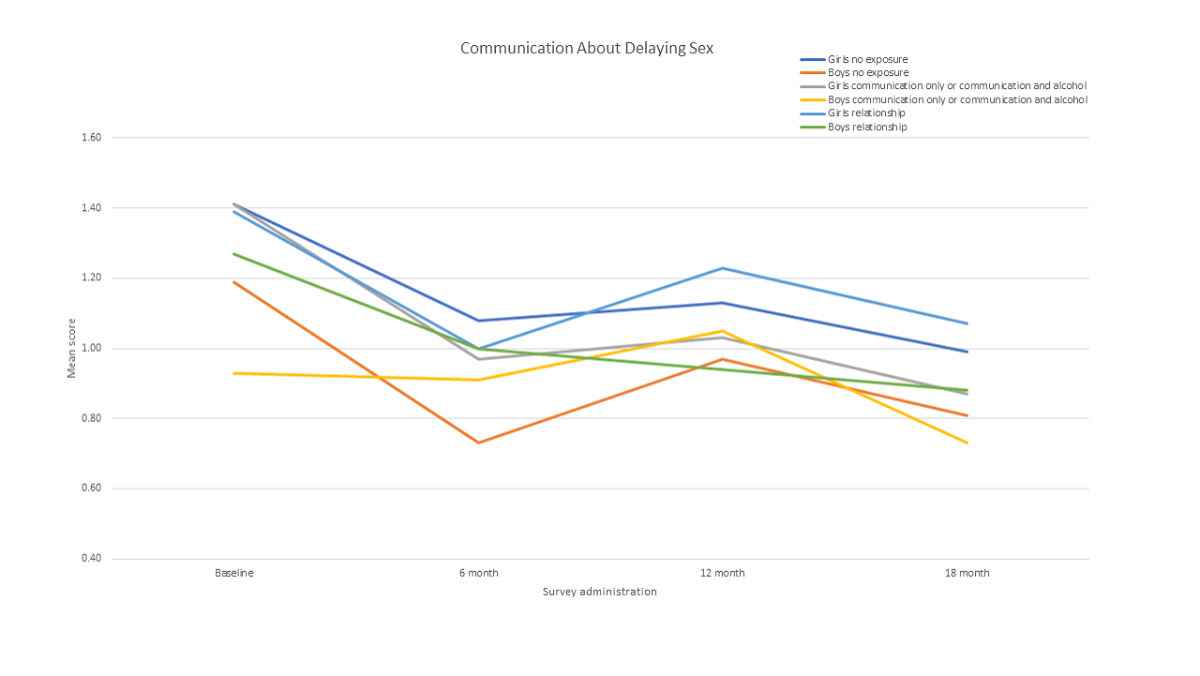
Gender by exposure over time (mean scores).

Three interaction effects were added to assess the impact of the intervention over time and by gender: (1) intervention by survey administration time, (2) intervention by gender, and (3) intervention by time by gender (data not shown). There were indications of an impact of the intervention over time (regardless of gender), with higher reports of several communication outcomes. At 6 months, adolescents in the intervention group reported more communication about delaying sex (B*=*0.27; SE 0.14; 95% CI −0.004 to 0.54; *P*=.05) and health risk communication (B*=*0.22; SE 0.11; 95% CI 0.01-0.44; *P*=.04). At 12 months, they reported more communication about the provision of birth control (B*=*0.70; SE 0.36; 95% CI −0.01 to 1.41; *P*=.05). Only one communication outcome showed some differential impact of the intervention over time by gender: as noted in Figure 2, the lighter blue line represents girls exposed to the relationships component, whose report of more communication regarding the delay of sexual behavior was significant at 12 months (B*=*−0.44; SE 0.22; 95% CI −0.87 to 0.01; *P*=.05).

## Discussion

### Principal Findings

The data support the efficacy of the Smart Choices 4 Teens intervention in increasing adolescent and parent communications about sexual relationships (eg, frequency) and increasing parental guidance (eg, dating rules). There was some support for our first hypothesis, in that the intervention had a main effect on frequency of communication and dating rules set for the adolescents, even after controlling for other adolescent characteristics that were also significant predictors of adolescents’ reports of sexual communication with their parents, including developmental changes over time, as well as adolescent gender, sexual minority status, and sexual experience.

There was some evidence to suggest that the intervention impacts parent-adolescent communication based on the gender of the adolescent. In general, girls are more likely than boys to report more frequent parent-adolescent conversations, and there was some evidence that the intervention had a greater long-term impact on increasing conversations about delaying sex with girls. One possible explanation may be that parents either consciously or unconsciously consider adolescent girls to be more responsible for sexual decision making than boys. Another possible explanation is that mothers are engaged in more conversations with adolescents than fathers; parent-adolescent communication about sexual activity may be more *comfortable* when conducted with same-sex combinations.

However, there were more indications that the intervention had some impact on sexual communication over time, irrespective of the gender of the adolescent. When accounting for the possible interaction between the time of administration, gender of the adolescent, and exposure to the relationships component, the effect of the relationships component over time (and not by gender) was more often the one that demonstrated an impact on the communication outcome. Throughout the literature on adolescent sexuality, although many sexual outcomes and measures of sexual communication often vary by gender, we found that this intervention had an impact on adolescents regardless of their gender. The intervention increased the number of sexual communication reports from both boys and girls, even in terms of dating rules, which also contained items that indicated the greater parental monitoring of adolescent relationships. There was no indication of an interaction effect between gender and intervention exposure on dating rules. Previous research indicates that girls (and parents of girls) tend to report greater relationship monitoring, as indicated by a higher number of dating rules. However, participating in the intervention increased the dating rules for all participants and was durable across time.

Findings from the study are limited by the overall number of families that engaged in the relationship component and the ability of the probit model to capture their most salient features [62]. The number of families completing the program also limited the ability to test differences in outcomes for adolescents who represent sexual and ethnic minorities. Replication studies that allow a more extensive examination of program effects are needed. Given that significant results were found with a reduced number of families, future replication studies that address engagement may find more robust support for the program. Forcing families to complete the two other components before accessing the relationships component contributed to the reduced completion rate for families in this component. Additional trials are needed to replicate these findings, and these efforts need to address engagement efforts that may help sustain families in completing the intervention. Future studies may also allow families to choose the order of completing the components. Engaging families in prevention programs requires overcoming the perception of many parents that their children do not need prevention programs because they have not observed any problems in the targeted areas.

Developing a universal prevention program for families that addresses adolescent relationships requires balancing the divergent views and needs of families. By allowing adolescents and parents to separately negotiate the program, this program allowed some self-determination in covering the materials and the amount of time they spent on the various elements of the program. Providing families with four different discussion scenarios and guiding questions meant that families had some element of choice in deciding what was most relevant to their family. Future replications of this program could enhance these discussion topics to address current issues that emerge in the community (eg, emerging challenges regarding casual sexual hook-ups for adolescents to negotiate). Future replications could also embed updated factual information into existing program elements (eg, state-specific rates and types of sexually transmitted infections for adolescents). Finally, the sexual and gender diversity of the adolescents were not examined in depth to further disentangle the impact of the program on specific adolescents. Gender was measured as a binary construct, which has decreasing relevance over time, as many adolescents have chosen to identify ways that defy such rigid definitions. Future implementation and assessment of this intervention will ensure that nonbinary gender identification choices are permitted. Although the measure of sexual diversity was inclusive, the sample size and space limitations did not permit more detailed analyses to understand nuance beyond strictly heterosexual or not.

Beyond the ability to adapt a web-based program for families, other advantages for building on an existing web-based intervention are that the costs for web-based delivery are relatively small and the reach is potentially large. Engaging families and making web-based programs accessible are important issues to be addressed in future research. Such programs can complement existing program efforts in schools and health care settings.

### Conclusions

The Smart Choices 4 Teens intervention was designed to be sensitive to the transition to young adulthood and increased independent decision-making. The intervention addresses critical needs for families of older adolescents, including the perspectives of adolescents and parents, leveraging the continued influence of parents even later in adolescence, and permitting flexibility to the schedules of families. Most importantly, this intervention shows promise for long-term impact on increasing parent-adolescent sexual communication, potentially providing some skills and capacity to handle the relationship demands of older adolescents.

## Appendix

Multimedia Appendix 1 CONSORT (Consolidated Standards of Reporting Trials)-eHEALTH checklist (version 1.6.1).

## Abbreviations

CONSORT: Consolidated Standards of Reporting Trials
IMR: inverse Mills ratio

## References

[1] Burgess V, Dziegielewski S, Green C (2005). Improving comfort about sex communication between parents and their adolescents: practice-based research within a teen sexuality group. Brief Treat Crisis Interven.

[2] Pariera KL, Brody E (2017). “Talk More About It”: Emerging adults’ attitudes about how and when parents should talk about sex. Sex Res Soc Policy.

[3] Ashcraft AM, Murray PJ (2017). Talking to parents about adolescent sexuality. Pediatr Clin North Am.

[4] Diiorio C, Pluhar E, Belcher L (2009). Parent-child communication about sexuality. J HIV/AIDS Prev Edu Adol Child.

[5] Bleakley A, Khurana A, Hennessy M, Ellithorpe M (2018). How patterns of learning about sexual information among adolescents are related to sexual behaviors. Perspect Sex Reprod Health.

[6] Coakley TM, Randolph S, Shears J, Beamon ER, Collins P, Sides T (2017). Parent-youth communication to reduce at-risk sexual behavior: a systematic literature review. J Hum Behav Soc Environ.

[7] Balaji AB, Oraka E, Fasula AM, Jayne PE, Carry MG, Raiford JL (2017). Association between parent-adolescent communication about sex-related topics and HIV testing, United States. 2006-2013. AIDS Care.

[8] Widman L, Choukas-Bradley S, Noar SM, Nesi J, Garrett K (2016). Parent-adolescent sexual communication and adolescent safer sex behavior: a meta-analysis. JAMA Pediatr.

[9] Haglund KA, Fehring RJ (2010). The association of religiosity, sexual education, and parental factors with risky sexual behaviors among adolescents and young adults. J Relig Health.

[10] Hahm H, Lee J, Zerden L, Ozonoff A, Amodeo M, Adkins C (2007). Longitudinal effects of perceived maternal approval on sexual behaviors of Asian and Pacific Islander (API) young adults. J Youth Adolescence.

[11] Negy C, Velezmoro R, Reig-Ferrer A, Smith-Castro V, Livia J (2016). Parental influence on their adult children's sexual values: a multi-national comparison between the United States, Spain, Costa Rica, and Peru. Arch Sex Behav.

[12] Simons LG, Burt CH, Tambling RB (2012). Identifying mediators of the influence of family factors on risky sexual behavior. J Child Fam Stud.

[13] Maria DS, Markham C, Bluethmann S, Mullen PD (2015). Parent-based adolescent sexual health interventions and effect on communication outcomes: a systematic review and meta-analyses. Perspect Sex Reprod Health.

[14] Blake SM, Simkin L, Ledsky R, Perkins C, Calabrese JM (2001). Effects of a parent-child communications intervention on young adolescents' risk for early onset of sexual intercourse. Fam Plan Perspect.

[15] Marseille E, Mirzazadeh A, Biggs MA, Miller AP, Horvath H, Lightfoot M, Malekinejad M, Kahn JG (2018). Effectiveness of school-based teen pregnancy prevention programs in the USA: a systematic review and meta-analysis. Prev Sci.

[16] Manlove J, Fish H, Moore KA (2015). Programs to improve adolescent sexual and reproductive health in the US: a review of the evidence. Adolesc Health Med Ther.

[17] Downing J, Jones L, Bates G, Sumnall H, Bellis MA (2011). A systematic review of parent and family-based intervention effectiveness on sexual outcomes in young people. Health Educ Res.

[18] Mytton J, Ingram J, Manns S, Thomas J (2014). Facilitators and barriers to engagement in parenting programs: a qualitative systematic review. Health Educ Behav.

[19] Allison S, Bauermeister JA, Bull S, Lightfoot M, Mustanski B, Shegog R, Levine D (2012). The intersection of youth, technology, and new media with sexual health: moving the research agenda forward. J Adolesc Health.

[20] Hadley W, Brown LK, Barker D, Warren J, Weddington P, Fortune T, Juzang I (2016). Work It Out Together: Preliminary efficacy of a parent and adolescent DVD and workbook intervention on adolescent sexual and substance use attitudes and parenting behaviors. AIDS Behav.

[21] Bayley JE, Brown KE (2015). Translating group programmes into online formats: establishing the acceptability of a parents' sex and relationships communication serious game. BMC Public Health.

[22] Gilliam M, Jagoda P, Jaworski E, Hebert LE, Lyman P, Wilson MC (2015). “Because if we don’t talk about it, how are we going to prevent it?”: lucidity, a narrative-based digital game about sexual violence. Sex Edu.

[23] Wurdak M, Kuntsche E, Wolstein J (2016). Effectiveness of an email-based intervention helping parents to enhance alcohol-related parenting skills and reduce their children’s alcohol consumption – a randomised controlled trial. Drug Educ Prev Polic.

[24] Pot M, Paulussen TG, Ruiter RA, Eekhout I, de Melker HE, Spoelstra ME, van Keulen HM (2017). Effectiveness of a web-based tailored intervention with virtual assistants promoting the acceptability of HPV vaccination among mothers of invited girls: randomized controlled trial. J Med Internet Res.

[25] Cornelius JB, Cato M, Lawrence JS, Boyer CB, Lightfoot M (2011). Development and pretesting multimedia HIV-prevention text messages for mobile cell phone delivery. J Assoc Nurses AIDS Care.

[26] Cornelius J, Dmochowski J, Boyer C, Lawrence JS, Lightfoot M, Moore M (2013). Text-messaging-enhanced HIV intervention for African American adolescents: a feasibility study. J Assoc Nurses AIDS Care.

[27] Jones K, Williams J, Sipsma H, Patil C (2019). Adolescent and emerging adults' evaluation of a Facebook site providing sexual health education. Public Health Nurs.

[28] Sun WH, Wong CK, Wong WC (2017). A peer-led, social media-delivered, safer sex intervention for Chinese college students: randomized controlled trial. J Med Internet Res.

[29] Drost RM, Paulus AT, Jander AF, Mercken L, de Vries H, Ruwaard D, Evers SM (2016). A web-based computer-tailored alcohol prevention program for adolescents: cost-effectiveness and intersectoral costs and benefits. J Med Internet Res.

[30] (2021). Internet/broadband fact sheet. Pew Research Center.

[31] Byrnes HF, Miller BA, Grube JW, Bourdeau B, Buller DB, Wang-Schweig M, Woodall WG (2019). Prevention of alcohol use in older teens: a randomized trial of an online family prevention program. Psychol Addict Behav.

[32] Craig BM, Hays RD, Pickard AS, Cella D, Revicki DA, Reeve BB (2013). Comparison of US panel vendors for online surveys. J Med Internet Res.

[33] Wang-Schweig M, Miller BA, Buller DB, Byrnes HF, Bourdeau B, Rogers V (2017). Using panel vendors for recruitment into a web-based family prevention program: methodological considerations. Eval Health Prof.

[34] Bauman KE, Foshee VA, Ennett ST, Hicks K, Pemberton M (2001). Family matters: a family-directed program designed to prevent adolescent tobacco and alcohol use. Health Promot Pract.

[35] Abar C, Turrisi R (2008). How important are parents during the college years? A longitudinal perspective of indirect influences parents yield on their college teens' alcohol use. Addict Behav.

[36] Ausubel D, Montemayor R, Svajian P (1977). Theory and Problems of Adolescent Development.

[37] Clausen JA (1968). Socialization and Society.

[38] Foshee V, Bauman KE (1992). Parental and peer characteristics as modifiers of the bond-behavior relationship: an elaboration of control theory. J Health Soc Behav.

[39] Hirschi T (2001). Causes of Delinquency.

[40] Hawkins JD, Weis JG (1985). The social development model: an integrated approach to delinquency prevention. J Primary Prevent.

[41] Brook J, Brook D, Gordon A, Whiteman M, Cohen P (1990). The psychosocial etiology of adolescent drug use: a family interactional approach. Genet Soc Gen Psychol Monogr.

[42] Bandura A (1976). Social Learning Theory.

[43] Glanz K, Lewis F, Rimer B (1991). The scope of health promotion and health education. Health Behavior and Health Education: Theory, Research, and Practice.

[44] Maibach E, Parrott R (1995). Designing Health Messages: Approaches from Communication Theory and Public Health Practice.

[45] O'Keefe D (1990). Persuasion Theory and Research.

[46] Turrisi R, Jaccard J, Taki R, Dunnam H, Grimes J (2001). Examination of the short-term efficacy of a parent intervention to reduce college student drinking tendencies. Psychol Addict Behav.

[47] Teitelman AM, Loveland-Cherry CJ (2005). Girls' perspectives on family scripts about sex-related topics and relationships. J HIV/AIDS Prev Child Youth.

[48] Basile KC, Rostad WL, Leemis RW, Espelage DL, Davis JP (2018). Protective factors for sexual violence: understanding how trajectories relate to perpetration in high school. Prev Sci.

[49] Dittus PJ, Michael SL, Becasen JS, Gloppen KM, McCarthy K, Guilamo-Ramos V (2015). Parental monitoring and its associations with adolescent sexual risk behavior: a meta-analysis. Pediatrics.

[50] Ethier KA, Harper CR, Hoo E, Dittus PJ (2016). The longitudinal impact of perceptions of parental monitoring on adolescent initiation of sexual activity. J Adolesc Health.

[51] Cupp PK, Atwood KA, Byrnes HF, Miller BA, Fongkaew W, Chamratrithirong A, Rhucharoenpornpanich O, Rosati MJ, Chookhare W (2013). The impact of Thai family matters on parent-adolescent sexual risk communication attitudes and behaviors. J Health Commun.

[52] Turrisi R, Abar C, Mallett K, Jaccard J (2010). An examination of the mediational effects of cognitive and attitudinal factors of a parent intervention to reduce college drinking. J Appl Soc Psychol.

[53] Reimuller A, Hussong A, Ennett ST (2011). The influence of alcohol-specific communication on adolescent alcohol use and alcohol-related consequences. Prev Sci.

[54] Madsen SD (2008). Parents’ management of adolescents’ romantic relationships through dating rules: gender variations and correlates of relationship qualities. J Youth Adolescence.

[55] Feinstein BA, Thomann M, Coventry R, Macapagal K, Mustanski B, Newcomb ME (2018). Gay and bisexual adolescent boys' perspectives on parent-adolescent relationships and parenting practices related to teen sex and dating. Arch Sex Behav.

[56] Sexual Minority Assessment Research Team (2009). Best practices for asking questions about sexual orientation on surveys. The Williams Institute of the University of California Los Angeles School of Law.

[57] Heckman JJ (1979). Sample selection bias as a specification error. Econometrica.

[58] Certo ST, Busenbark JR, Woo H, Semadeni M (2016). Sample selection bias and Heckman models in strategic management research. Strat Mgmt J.

[59] Tucker JW (2010). Selection bias and econometric remedies in accounting and finance research. J Account Lit.

[60] Gross D, Fogg L (2004). A critical analysis of the intent-to-treat principle in prevention research. J Prim Prev.

[61] Coie JD, Watt NF, West SG, Hawkins JD, Asarnow JR, Markman HJ, Ramey SL, Shure MB, Long B (1993). The science of prevention: a conceptual framework and some directions for a national research program. Am Psychol.

[62] Titus MA (2006). Detecting selection bias, using propensity score matching, and estimating treatment effects: an application to the private returns to a master’s degree. Res High Educ.

